# “Breaking the bars of my prison of fear” a trauma- and resilience-informed photovoice model as a community intervention following a human-made collective disaster

**DOI:** 10.3389/fpsyg.2026.1948831

**Published:** 2026-10-06

**Authors:** Menny Malka

**Affiliations:** Ben-Gurion University of the Negev, Be'er Sheva, Israel

**Keywords:** collective trauma, community recovery, photovoice, resilience narratives, trauma-informed practice

## Abstract

**Introduction:**

Human-made collective disasters disrupt not only physical safety but also the systems of meaning, relationships, and shared narratives that sustain community life. This article presents a Trauma- and Resilience-Informed Photovoice Model (TRIPVM) as an arts-based community intervention and illustrates its implementation in the Israeli city of Ofakim following the October 7, 2023 terrorist attack.

**Methods:**

Developed within a Community-Based Participatory Research (CBPR) framework, the model integrates principles of disaster management, trauma- and resilience-informed practice, and the visual arts to support community recovery. The intervention involved 11 community groups facilitated by 22 trained facilitators and combined photography, narrative writing, structured group dialogue, and a public exhibition.

**Results:**

The article describes the model's theoretical foundations, implementation protocol, and practical application through representative examples from the intervention. The model is organized around four complementary principles: (1) constructing resilience narratives; (2) strengthening community recognition and ownership of collective narratives; (3) identifying and mobilizing individual and community coping resources; and (4) fostering agency, choice, and the reframing of traumatic experiences through artistic expression.

**Discussion:**

The proposed model extends Photovoice beyond its traditional role in participatory research by offering a practical, arts-based framework for community recovery following war, terrorism, and other human-made collective disasters.

## 1 Introduction

The consequences of collective disasters, particularly human-made ones, extend beyond physical destruction and loss of life, undermining individual and community safety, psychological stability, and the social meanings attached to life within the affected community (Centemeri and Tomassi, 2022). Beyond their immediate impact, such disasters disrupt daily routines, erode feelings of security, and generate distress and trauma at both the individual and community levels (Farny et al., 2025; Ngulube, 2024). At the same time, they often give rise to processes of adaptation, reorganization, and meaning-making supported by institutional responses, local initiatives, and existing support networks (Jordan-Martin et al., 2020; Rawsthorne et al., 2023). This combination of disruption and adaptation has led disaster research and practice to increasingly recognize that recovery extends beyond individual psychological treatment and requires community-based interventions (Ersing, 2020; Itzhaki-Braun et al., 2023; Saul, 2022).

Within this broader focus on community-based recovery, narrative interventions have gained particular attention because they create shared spaces to process traumatic experiences, construct collective narratives, foster commemorative practices, and generate shared meaning following disasters (Sanderson et al., 2024). Their value is grounded in theoretical perspectives that view narratives, representations, and community practices as central to processing collective trauma, organizing experience, shaping individual and collective identities, and fostering collective action (Ganz, 2009; Sanderson et al., 2024; Seeger and Sellnow, 2016; Singh et al., 2026; Yamori, 2025).

However, communities do not have equal opportunities to construct and represent their narratives. Critical perspectives further emphasize that community representation and commemorative practices are embedded in power relations and structural inequalities, creating tensions between local narratives and dominant public discourses; between communities‘ claims to recognition and ownership of their stories, and others' appropriation of those stories (Maciel and Sellnow, 2025; Milošević, 2024; Mosley and Biernat, 2025). These tensions are also reflected in the commodification of trauma and the transformation of disaster-affected communities into sites of memory (Stone and Grebenar, 2022) and may be particularly significant for communities that were already marginalized or peripheral prior to a disaster.

Indeed, a broad body of evidence indicates that communities that were marginalized or peripheral prior to a disaster enter it with pre-existing structural vulnerabilities that continue to shape their experiences of the disaster and their capacity for recovery (e.g., Ferreira et al., 2024; Few et al., 2023; Hamideh, 2020; Joseph et al., 2021; Matthewman and Uekusa, 2021; Pilogallo et al., 2025). For example, a study based on two case studies among marginalized communities in India showed that recovery processes are shaped by inequalities, social relations, and material conditions that existed prior to the disaster, and that formal recovery mechanisms may overlook these conditions, thereby reproducing existing marginalization (Joseph et al., 2021). Similarly, a study of peripheral inner areas in the Abruzzo region of Italy found that pre-existing conditions of marginalization including depopulation, geographical isolation, economic stagnation, and limited infrastructure, exacerbated the impacts of earthquakes and constrained the capacity of smaller municipalities to access and utilize recovery resources (Pilogallo et al., 2025).

These inequalities extend beyond material and institutional vulnerability and affect communities' capacity to voice their concerns and influence how recovery is defined and represented. A scoping review of 108 studies from the United States found that historically marginalized communities face limited access to political power and decision-making processes, and that narratives produced by politicians and the media may shape perceptions of communities' viability and deservingness and, consequently, the allocation of recovery resources (Ferreira et al., 2024). These findings are consistent with a qualitative study conducted following Hurricane Ike in Texas, which showed that groups marginalized prior to the disaster were substantially underrepresented in recovery planning and that pre-existing social divisions and historical distrust limited their ability to voice their needs and influence recovery priorities (Hamideh, 2020).

The importance of representation and voice is also evident in research conducted across disaster-affected communities in three states in India, which found that dominant narratives of disaster and recovery can obscure pre-existing inequalities and render the voices and needs of historically marginalized communities less visible. The authors therefore emphasize the importance of spaces for self-representation through which marginalized communities can articulate their own needs and perspectives (Few et al., 2023). At the theoretical level, a synthesis of disaster research highlights the role of symbolic capital in these processes: communities with greater social standing, visibility, and recognition are more likely to receive positive public attention and external support, whereas communities with lower symbolic capital or those subject to stigma face greater difficulties in gaining recognition and support during recovery (Matthewman and Uekusa, 2021).

Thus, these findings underscore the importance of narrative ownership and self-representation in disaster recovery, particularly for communities that were already marginalized prior to the disaster. One arts-based methodology strongly associated with addressing power relations, fostering community partnerships, documenting and amplifying community knowledge, and supporting community ownership of their narratives is Photovoice (Wang and Burris, 1997). Since its development in the 1990s, substantial evidence has demonstrated its capacity to enable community members to construct their stories through individual, group, and collective processes, facilitating the shift from personal to collective narratives (Malka and Rahat-Driham, 2025). Recent studies further show that Photovoice in trauma contexts enables expression of complex experiences, supports emotional processing, reinforces feelings of belonging and solidarity, and may contribute to collective meaning-making in the public sphere (Dixon et al., 2022; Olausson and Lindahl, 2022). However, in disaster and trauma settings, Photovoice has largely been used as a research tool, often implemented primarily for data collection (Bray et al., 2023), rather than as an integrated community process combining research and intervention (Malka and Lotan, 2023, 2025).

To address this gap, the present paper introduces a Trauma- and Resilience-Informed Photovoice Model (TRIPVM), developed and implemented as a community intervention in the city of Ofakim following the October 7, 2023, terrorist attack in the Mishor HaGefen neighborhood of Ofakim. The exhibition *Our Story, Ofakim* (Figure 1), presented in September 2024, marked the culmination of the process, illustrating a transition from individual experience and expression to collective public visibility.

**Figure 1:**
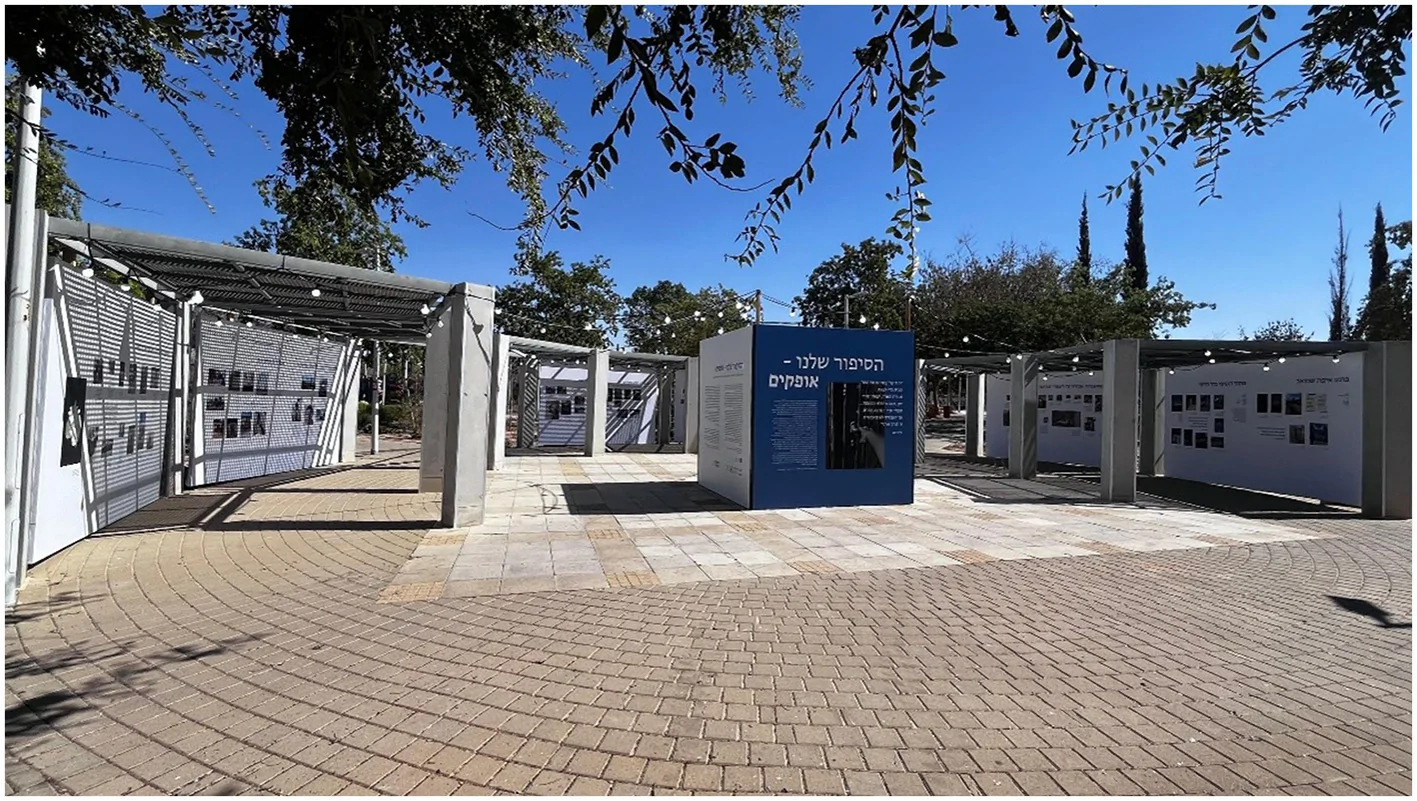
The photovoice exhibition our story - Ofakim (photograph by IBD, September 2024).

To situate the applied model within the theoretical and social context of its implementation, the article first reviews the literature on collective disasters and Photovoice. It then presents TRIPVM's theoretical and practical principles, followed by a description of the Ofakim context, the broader post–October 7 intervention, and TRIPVM's implementation, including representative examples. The paper concludes by discussing key insights and principles that emerged from the intervention.

## 2 Literature review

The literature review addresses two bodies of knowledge that inform the proposed model. First, it examines collective disasters as traumatic events, with attention to their psychosocial and community consequences and to processes of recovery and meaning making. Second, it reviews Photovoice as a participatory approach and considers its potential in the context of trauma and community recovery. Together, these perspectives provide the conceptual foundation for the Trauma- and Resilience-Informed Photovoice Model (TRIPVM).

### 2.1 Collective disaster as a traumatic event

A collective disaster is described in the literature as a large-scale, often sudden and unexpected event that causes substantial damage, destruction, and human suffering (Perry, 2018). Such events disrupt ordinary community or societal life and create conditions in which the scale of loss and distress exceeds local coping capacities, often requiring external assistance (Bonanno et al., 2010). Accordingly, collective disasters are characterized as an extreme disruption to social functioning that results in significant human, material, and economic losses [Mitchell, 2021; United Nations Office for Disaster Risk Reduction (UNODRR), 2017]. Beyond physical destruction, disasters undermine social systems, reshape social relations, and damage collective security (Centemeri and Tomassi, 2022), thereby creating conditions for distress and trauma at both the individual and community levels (Farny et al., 2025; Ngulube, 2024). Importantly, however, disaster and trauma are distinct: disaster refers to the event itself, whereas trauma refers to its psychosocial consequences (Heanoy and Brown, 2024). As (Weik 2008) argued, the connection between the two creates conditions in which trauma can fracture and destabilize existing meanings.

At the community level, these consequences extend beyond individual experiences of trauma. In collective disasters, traumatic, social, and physical effects are not confined to individuals but extend across the physical, social, and psychological dimensions of the entire community (Flores et al., 2025). Within this broader understanding of collective disasters, disaster research distinguishes between types of disasters, particularly between natural and human-made events (Quarantelli, 1998; Su et al., 2022).

Natural disasters often involve widespread infrastructural damage and displacement, while also revealing underlying social vulnerabilities (Klinenberg, 2015). In contrast, human-made disasters tend to produce prolonged social disruption and more intense collective trauma (Longman, 2017). Studies have also identified differences in psychosocial outcomes and recovery processes across disaster types. Natural disasters may be associated with faster community mobilization and broader institutional involvement in recovery processes (Sanderson et al., 2024), whereas human-made disasters are often associated with higher levels of distress and post-traumatic symptoms, partly due to the erosion of trust and social cohesion (Makwana, 2019). Consistent with these distinctions, psychological distress and the erosion of public trust are often intensified in human-made disasters (Bonanno et al., 2010), in contrast to natural disasters, which may generate more rapid community mobilization (Su et al., 2022).

Evidence regarding these differences, however, is not unequivocal. In a systematic review of 284 reports on post-disaster PTSD, (Neria et al. 2008) found that PTSD prevalence following natural disasters was generally lower than that reported after human-made and technological disasters, while cautioning that differences in the severity and proximity of exposure may partly account for this pattern. Similarly, using data from the World Mental Health Surveys, (Bromet et al. 2017) found that human-made disasters were associated with significantly higher odds of PTSD than natural disasters among respondents from high-income countries, although this association became less pronounced after accounting for disaster-related characteristics.

More recently, a systematic review and multilevel meta-analysis of 71 longitudinal studies comprising 137,004 participants found no significant differences in the prevalence of mental health problems between natural and human-made disasters after controlling for multiple factors (Dückers et al., 2026). Taken together, these findings suggest that although human-made disasters may in some circumstances be associated with more severe psychosocial consequences, these differences cannot be attributed to disaster type alone and need to be understood in relation to the nature and severity of exposure and the vulnerability profiles of affected populations. Across disaster types, research also points to processes of resilience and post-traumatic growth (Dückers et al., 2026).

In both cases, disasters not only create new vulnerabilities but also expose preexisting community challenges, deepen existing social inequalities, and exacerbate harm among marginalized communities (Van Dorn et al., 2020). Studies nevertheless show that communities do not remain in a static state of crisis but undergo processes of recovery, adaptation, and reorganization (Aung et al., 2021; Ersing, 2020; Rawsthorne et al., 2023). Some emphasize that recovery and reconstruction are not limited to rebuilding infrastructure but also involve reshaping social relations, renegotiating collective identity, and constructing shared meanings around the disaster (Singh et al., 2026; Weik, 2008).

Accordingly, growing attention has been given to community practices for processing disaster (Sherwood et al., 2024), including the construction of post-disaster community narratives, the cultivation of collective memory, and commemorative practices (Singh et al., 2026). These practices enable communities to organize experiences of rupture and construct meaning; when they do not function merely as a placebo (Milošević, 2024), they may contribute significantly to recovery and healing. Moreover, given the traumatic nature of collective disasters, scholars have emphasized critical practices that foreground knowledge production through community members' perspectives and contribute to shared discourse, belonging, control, and agency (Catalani and Minkler, 2010; Hou and Hearn, 2026).

### 2.2 Photovoice

Guided by the foundational principle of “nothing about us without us” (Charlton, 1998), Photovoice is a critical community-based participatory research methodology developed in the 1990s that combines knowledge production, community empowerment, and social action (Wang and Burris, 1997). It rests on the assumption that community members are experts in their own lives and that, through visual and narrative tools, they can document, interpret, and make visible their experiential knowledge. In doing so, Photovoice challenges traditional hierarchies of knowledge production and positions participants as active partners in interpreting reality and constructing meaning while linking lived experience to broader social contexts (Anderson et al., 2023; Catalani and Minkler, 2010). These principles are grounded in a theoretical foundation that combines critical pedagogy, feminist theory, and visual research, emphasizing dialogue, reflexivity, and recognition of knowledge grounded in lived experience (Wang and Burris, 1997).

On this basis, the central aims of Photovoice include: (1) to document and reflect on community strengths and concerns; (2) to promote critical dialogue through group discussion of photographs; and (3) to foster action by raising awareness and engaging decision-makers through a community exhibition (Bray et al., 2023; Wang and Burris, 1997). These aims structure a gradual process in which photography provides the starting point, group dialogue enables meaning-making, and public visibility links knowledge with action. This facilitates a movement from the “Story of Self” to the “Story of Us,” providing a basis for understanding shared reality and promoting collective action (Ganz, 2009).

Regarding the combination of photography and narrative (photovoice), while the photograph provides a concrete anchor for documenting lived experience through “freezing phenomenological moments” in time and space and transporting them into the group and community arena (Malka, 2022), the accompanying narrative enables these moments to be situated and analyzed within social, cultural, and political contexts. Moreover, the integration of visual imagery and group processing enables a shift from personal reflection to collective meaning-making and the development of shared critical consciousness (Anderson et al., 2023). Practically, narrative writing is often guided by Wang's (1999) structured SHOWeD protocol, which comprises five questions: what is Seen in the photograph; what is really Happening; how this relates to Our lives as a community; Why the social conditions or challenges represented exist; and what can be Done. The SHOWeD protocol thus serves as an organizing framework that helps translate personal experience into collective understanding and collective action within a group space.

In recent years, Photovoice has been increasingly used with vulnerable populations, particularly those exposed to trauma (Katz et al., 2024; Tan et al., 2024). Studies have highlighted its advantages in creating channels for expressing complex experiences that are often difficult to articulate verbally, while supporting emotional processing, meaning-making, and belonging (Dixon et al., 2022; Olausson and Lindahl, 2022; Pichon et al., 2022). A smaller body of studies has demonstrated partial applications of Photovoice in contexts of collective trauma, emphasizing its value in creating a safe space for participants, allowing them greater control over what they share, and constructing meaning around disaster events (Bray et al., 2023; Cecen Celik et al., 2026; de la Roche et al., 2025; Padilla et al., 2024).

Building on this background, the present paper aims to present a TRIPVM in the context of a human-made collective disaster, as an adaptation of the classic Photovoice model for disaster intervention. It is illustrated through the implementation in the city of Ofakim following the events of October 7 and in relation to the broader municipal intervention. The article outlines its theoretical, professional, and practical principles, together with illustrative examples and key insights for future implementation.

### 2.3 The intervention model: a trauma- and resilience-informed photovoice model

TRIPVM, developed as a community intervention following a collective disaster, is built on four core components: (1) *Disaster Management* as a framework for understanding post-disaster intervention; (2) *Resilience Narratives*, emphasizing the role of narrative in post-disaster meaning-making; (3) *Recognition and Ownership of the Story*, supporting the construction of a collective narrative from the community's perspective; and (4) *Adaptation of the Photovoice Facilitation Protocol*, modifying the standard Photovoice facilitation protocol for contexts of disaster, trauma, and resilience.

#### 2.3.1 Photovoice as a community intervention within the disaster management framework

TRIPVM was implemented as part of a broader community intervention designed to provide a structured setting in which community members can engage in guided supported reflection and processing while strengthening connections to coping resources, enhancing ownership of their story, and supporting the construction of a shared narrative. The intervention is situated within the **Relief–Rehabilitation–Development (RRD)** model of disaster management (Berke and Campanella, 2006; Christoplos et al., 2001), which conceptualizes post-disaster recovery as three interrelated stages (Figure 2).

**Figure 2:**
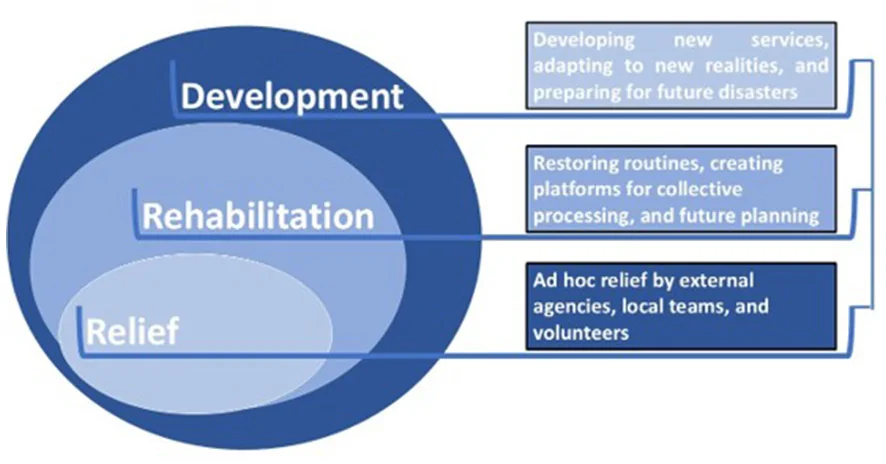
The relief–rehabilitation–development (RRD) disaster management model.

##### 2.3.1.1 Relief

Immediate response focused on meeting basic needs, saving lives, and ensuring physical safety and initial stabilization. This stage consists of short-term emergency actions drawing on both external assistance and internal community resources, providing the foundation for subsequent stages.

##### 2.3.1.2 Rehabilitation

Beyond restoring services and infrastructure, this stage includes community responses that facilitate processing of the disaster, establish new services, as well as memorial and commemorative frameworks, and construct shared meanings that support both a return to routine and the development of new routines.

##### 2.3.1.3 Development

Long-term interventions aimed at strengthening community resilience, reducing future vulnerabilities, and incorporating lessons learned.

These stages often overlap and are shaped by broader social, political, and economic contexts. Failure to transition effectively between them may perpetuate vulnerability, whereas successful integration enhances community resilience and preparedness for future disasters (Berke and Campanella, 2006; Christoplos et al., 2001). Within this framework, TRIPVM is primarily situated at the transition from relief to rehabilitation, where it supports processing of the disaster through resilience narratives grounded in community coping resources while strengthening community ownership and recognition of the shared story.

#### 2.3.2 Photovoice as a community intervention for documenting and disseminating resilience narratives

Building on the disaster management framework, TRIPVM is grounded in the premise that collective trauma disrupts the systems of meaning through which individuals and communities understand their world (Weik, 2008). Recovery therefore requires the reconstruction of meaning and collective memory, both of which are recognized as essential components of post-disaster recovery at the individual and community levels (Milošević, 2024). In community responses to disaster and war, the development of meaningful community narratives has likewise been identified as a key process (Sanderson et al., 2024; Seeger and Sellnow, 2016; Singh et al., 2026; Yamori, 2025).

Accordingly, this intervention draws on the concept of preferred or resilience narratives (Maung et al., 2021). Emphasizing such narratives has been identified not only as an effective strategy for reducing post-disaster risks but also as a sociopolitical stance that resists giving disproportionate weight to trauma-centered accounts of disaster (Imperiale and Vanclay, 2021). TRIPVM therefore seeks to document, generate, and disseminate resilience narratives following disaster and trauma (Rolbiecki et al., 2016). Because narratives organize events into coherent and meaningful wholes, they help individuals and communities make sense of post-disaster reality and act within it. Importantly, resilience narratives emerge through ongoing interactions between global discourses and local cultures, histories, and memories (Zada, 2026). They thus become resources for processing trauma, constructing meaning, and strengthening agency while remaining embedded in power relations shaped by the community's social position and symbolic capital (Fraile-Marcos, 2019). During the transition from relief to rehabilitation and development, resilience narratives function as collective stories that highlight local resources and shared beliefs, reinforce solidarity and collective action, and contribute to recovery (Basseler et al., 2020). They are also intertwined with practices of memory and commemoration, through which communities seek recognition of their loss and suffering while increasing their symbolic standing in ways that challenge negative public representations (Maciel and Sellnow, 2025; Milošević, 2024).

#### 2.3.3 Photovoice as a community intervention promoting recognition and ownership of the community story

Building on the discussion of resilience narratives, TRIPVM recognizes both the preexisting community vulnerabilities and those created by the disaster itself while seeking to strengthen community ownership over its representation and story. The importance of recognition and ownership has been emphasized both in critical scholarship on marginalized communities and disaster studies. Critical perspectives highlight the tension between communities' ability to tell their own stories from their own standpoint (*appropriated identity*) and externally imposed narratives shaped by prejudice and othering (*ascribed identity*). By democratizing knowledge production, Photovoice creates a platform through which communities can gain recognition and present their distinct perspectives, offering an alternative to both non-recognition and misrecognition (Mosley and Biernat, 2025; Taylor, 1994).

These concerns become especially salient following disasters within what has been termed the “*compassion industry*” (Polman, 2010). Communities directly affected by disasters often become public attractions and, at times, destinations for visitors, aid organizations, and the media, contributing to forms of dark or grief tourism. Such processes may undermine communities‘ control over how they are represented, how their stories are told, and how those stories are commercialized (Maciel and Sellnow, 2025; Mosley and Biernat, 2025; Stone and Grebenar, 2022). In this context, the concept of “*placebo memory*” (Milošević, 2024) critiques forms of post-disaster remembrance that overlook the needs and agency of community members while simultaneously placing them at the center of public attention. Such processes may ultimately undermine restorative justice, which depends in part on respect for and recognition of community members' lived experiences (Milošević, 2024).

Against this backdrop, TRIPVM seeks, through genuine partnership with community members, to create a space for documenting and constructing the community narrative while ensuring community ownership and control not only over individual stories but also over the collective narrative itself.

#### 2.3.4 Adapting the SHOWeD protocol and group facilitation for disaster, trauma, and resilience contexts

Alongside the critical foundations of the classic Photovoice approach, TRIPVM incorporates adaptations based on trauma- and resilience-informed practice at three levels: (1) the photography task and accompanying narrative; (2) group facilitation; and (3) public exhibition. The following section presents the adaptations at each level.

##### 2.3.4.1 Task-level adaptations: photography and narrative

The first adaptation concerns the photography prompt provided to the participants:

“*Photograph an object, place, or everyday situation that tells the world what it means to be a member of this community following the disaster, with particular emphasis on what gives me strength—that is, the coping resources of myself and my community”*.

To enhance participants‘ sense of control, they receive basic instruction in photographic techniques, including the rule of thirds, black-and-white photography, depth of field, composition, and the use of light. These skills increase intentionality and participants' sense of choice in image-making. Moreover, photography allows participants to document potentially triggering objects or situations in a deliberate and controlled manner.

A second adaptation concerns the accompanying narrative (“voice”), developed through a modified *SHOWeD* protocol, informed by Conservation of Resources Theory (Hobfoll, 2001):

**(S)** *See*: Describe what is visible in the photograph, paying attention to details, symbols, and visual elements, with particular emphasis on identifying visible and less visible coping resources; **(H)** *Happening*: Describe the story behind the image, connecting it to personal experiences of coping and adaptation; **(O)** *Our Lives*: Link the personal story to the broader community narrative, highlighting collective coping resources; **(W)** *Why*: Identify personal, interpersonal, and material sources of strength through the lens of Conservation of Resources Theory, supplemented by brief psychoeducational explanations and complementary models such as BASIC Ph (Lahad and Leykin, 2015) to deepen understanding of coping processes; **(D)** *Do*: Identify possible actions to preserve, strengthen, and expand the coping resources that emerged.

##### 2.3.4.2 Group facilitation principles

Group facilitation is guided by principles of trauma-informed group practice (Baird and Alaggia, 2021), adapted to the Photovoice methodology (Rolbiecki et al., 2016). These principles seek to maximize participants' control over the degree of disclosure and emotional engagement while reducing the risk of re-traumatization and supporting meaning-making and the transition from personal to collective narratives.

Five principles guide facilitation:

**(1)** *Sensitivity to disaster exposure*: Participants' exposure to the disaster and its traumatic impact is assessed in advance to understand their experiences, including bereavement and other significant losses; **(2)** *Creating safety and fostering respect*: The process begins with shared expectations and a group agreement that establishes emotional and physical safety through clear guidelines, optional breaks, and a nonjudgmental atmosphere; **(3)** *Supporting emotional regulation and self-care*: Orientation sessions introduce grounding and emotional regulation strategies early in the process to help participants manage emotional activation; **(4)** *Resource-oriented facilitation*: To reduce the risk of re-traumatization, facilitators use structured sharing rounds and guide discussion toward photographs, coping actions, and available resources. When participants spontaneously recount traumatic experiences, facilitators gently redirect attention toward action, meaning, and coping while allowing movement between pain and resilience; **(5)** *Sensitivity to power relations and marginalization*: Facilitation acknowledges the community's sociocultural position, experiences of injustice, shame, and limited symbolic capital, alongside its need for recognition.

To further promote psychological safety (Alessi and Kahn, 2023), both the photography assignments and the group meetings follow a structured format. Photography assignments focus on themes such as black-and-white photography, light and shadow, everyday life, and observing community spaces. Meetings include an opening round, structured sharing of photographs, an open discussion linking personal stories to the collective narrative, and a closing reflection.

##### 2.3.4.3 Public visibility: the exhibition as a practice of recognition and empowerment

The public exhibition plays a central role in post-disaster recovery by transforming private expression into public recognition. Presenting participants' work in public spaces creates opportunities for visibility, recognition, validation, meaning-making, and dialogue between communities, institutions, and policymakers (Bray et al., 2023; Padilla et al., 2024). It also brings personal narratives into broader public discourse, contributing to collective memory and strengthening community agency.

In TRIPVM, exhibition planning is guided by six principles:

**(1)** *Embedding the exhibition within a meaningful community context*: The timing and location of the exhibition are selected for their symbolic significance—for example, around the anniversary of the disaster—and may extend beyond the community itself to strategic venues of influence; **(2)** *Preparing participants from the outset*: Participants are informed from the outset that their work may be publicly exhibited and, whenever possible, about the anticipated timing and planning process. This encourages reflection on representation and intended audiences throughout the project; **(3)** *Participant control over exhibited materials*: Participants retain full authority over which photographs are displayed, ensuring control over both content and public exposure; **(4)** *Distinct group representation*: Where multiple groups participate (e.g., bereaved families, older adults), each group is given a dedicated exhibition space that reflects its unique identity, including a title and explanatory text representing its collective process and message; **(5)** *Exhibition preparation as part of the intervention*: Selecting, organizing, and preparing the exhibition is treated as an integral stage of the group process rather than a separate concluding activity; **(6)** *Voluntary public engagement*: Participants who wish to do so are invited to share their experiences and insights with visitors during the exhibition or gallery events. The opportunity to speak publicly contributes to recognition, empowerment, and a sense of collective voice (Figure 3).

**Figure 3:**
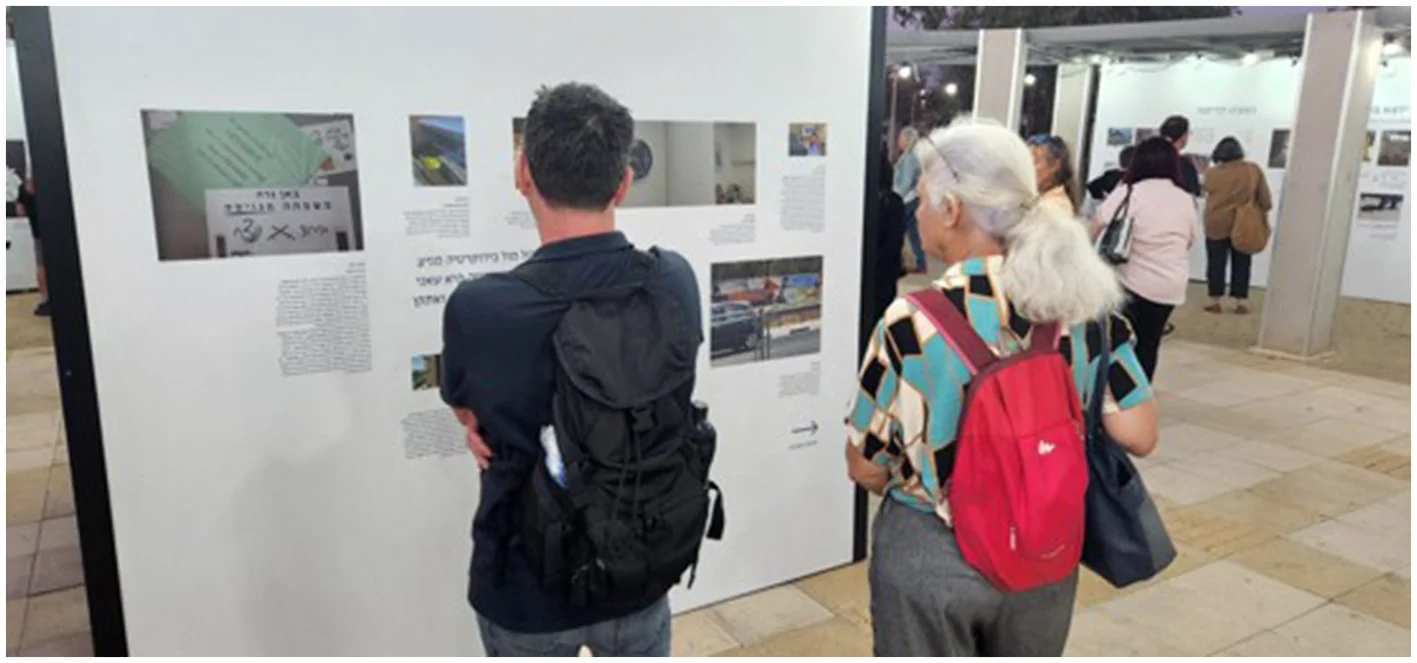
Visitors viewing a dedicated exhibition wall featuring the work of one of the photovoice groups (photograph by the author, September 2024).

## 3 Methodology

The present article presents an applied TRIPVM, developed and implemented as part of a large-scale community intervention following the October 7 events in the city of Ofakim. The model is grounded in the principles of Community-Based Participatory Research (CBPR), particularly the integration of knowledge production and systematic intervention (Catalani and Minkler, 2010). Accordingly, the model was developed, implemented, and conceptually refined through an engaged critical research approach (Malka, 2024), which views research, professional practice, and community knowledge as complementary and mutually informing processes.

### 3.1 Intervention context

Following the October 7 attacks, which constituted a human-made national collective disaster that severely undermined people's sense of security, trust in institutions, and core life systems (Gozlan et al., 2024; Neria et al., 2025), the author joined the team responsible for planning and implementing an emergency intervention for residents of the Mishor HaGefen neighborhood and the city of Ofakim. As the intervention evolved from the emergency phase toward rehabilitation, the author led the development of TRIPVMl, the training of facilitators, and the ongoing supervision of its implementation, integrating academic, professional, and community knowledge in accordance with CBPR principles (Catalani and Minkler, 2010).

### 3.2 Ofakim and the October 7 events

Established in 1955, Ofakim is recognized as both a development town and part of Israel's geographical and social periphery (Gigi and Tzfadia, 2024), characterized by longstanding socioeconomic challenges, immigration-related difficulties, and limited symbolic capital (Cohen, 2016), alongside recent processes of growth and demographic expansion (Central Bureau of Statistics (CBS), 2023). On the morning of October 7, militants infiltrated the city and operated within residential neighborhoods, primarily Mishor HaGefen, engaging in combat within an unprotected civilian environment. Thirty-three civilians were killed in the neighborhood, and an additional twenty residents were murdered outside the city and its surroundings. Beyond the devastating losses, the attacks brought unprecedented media attention to Ofakim, highlighting stories of local heroism while also reinforcing narratives of abandonment and the challenges faced by residents coping with persistent socioeconomic inequalities (Aharoni et al., 2024; Tversky, 2025). As illustrated later in the article, the city's historical and symbolic position, together with the tension between narratives of heroism and abandonment, played an important role in shaping the intervention and the development of community narratives.

### 3.3 Description of the intervention

On October 9, 2023, a municipal resilience center was established as the primary response for the city's residents through collaboration among professional agencies, providing emergency coordination while laying the groundwork for long-term intervention (Shahar et al., 2024). The intervention included complementary components: face-to-face support provided by social workers and psychologists, most of whom volunteered; proactive home visits (“door-to-door”); and telephone support that frequently led to continued care. In parallel, community resilience hubs operated alongside emergency teams and volunteers, supporting both civilians and first responders. This structure enabled the integration of individual services with community-based action while adapting to changing needs. Over time, the intervention gradually shifted from acute emergency response toward trauma processing and restoration of functioning, drawing on professional expertise, local teams, accumulated experience in disaster intervention and, above all, the recognition of the community's healing potential (Tversky, 2025).

As part of this long-term intervention, and in recognition of the need for collective processing of the October 7 events alongside residents' desire to tell their own story from their own perspective (Tversky, 2025), a large-scale Photovoice project was initiated. The project was also shaped by preparations for commemorating the first anniversary of the attacks and sought to provide a structured framework for individual and collective expression through photography, narrative writing, and group dialogue. The project was implemented in collaboration with academic institutions, the municipal Department of Social Services, the Joint Distribution Committee, Photo Israel, and Natan.

The intervention unfolded in five stages: (1) establishment of a steering committee responsible for defining the project's conceptual and organizational framework, with the community exhibition identified from the outset as the project's culminating event; (2) recruitment and training of facilitators, combining experiential learning with reflection and emphasizing the integration of visual language with trauma- and resilience-informed practice. Twenty-two facilitators participated, including 12 social workers and 10 Photo Israel facilitators, with each group jointly facilitated by a social worker and a Photovoice facilitator; (3) implementation of 11 diverse community groups between March and August 2024. Participants documented their experiences in response to the prompt: “*What would I like to tell the world about being a community member following the events of October 7?”*, with particular emphasis on everyday life and coping resources; (4) preparation of the community exhibition, including selection of photographs, formulation of key messages, and writing accompanying narratives for the photographs (Figures 1, 2). During this stage, participants collectively selected the title *Our Story, Ofakim*, reflecting their commitment to reclaiming the local narrative; and (5) presentation of the exhibition in September 2024 in a central public space, where it functioned not merely as the project's final product but as an integral component of the intervention, connecting individual, group, and community processes through collective expression, reflection, and meaning-making.

### 3.4 Data analysis

Implementation of TRIPVM was accompanied by a broader research program in which multiple sources of qualitative data were systematically collected and analyzed. The database comprised two primary units of analysis: (1) Photovoice units, consisting of photographs, titles, and accompanying narratives produced throughout the intervention; and (2) semi-structured, in-depth interviews with project participants exploring their experiences and the meanings they attributed to participation.

Interview data were analyzed using categorical thematic analysis (Lieblich et al., 2015), involving holistic reading, identification of recurring ideas, clustering into broader categories, and the development of overarching themes describing participants' experiences and interpretations of the intervention.

The Photovoice units were analyzed using a three-stage qualitative content analysis (Elo and Kyngäs, 2008) combined with a polytextual approach (Gleeson, 2012), treating each Photovoice unit as an integrated whole comprising the photograph, title, narrative, and broader social context. The analysis involved identifying meaning units, organizing them into subthemes, and generating overarching themes.

The findings from these analyses are reported elsewhere in two separate empirical publications. The present article draws on a secondary analysis of these datasets. However, rather than reporting empirical findings, its purpose is to present TRIPVM. Accordingly, the illustrative examples presented in the following sections were selected from the analyzed materials because they best demonstrate the model's central principles, components, and implementation. These examples should therefore be understood as analytic illustrations of the model rather than empirical findings in themselves.

### 3.5 Ethical considerations

The project and the accompanying studies received ethical approval from the Ethics Committee at the author's university. All participants provided written informed consent for participation in the project, public exhibition of selected materials, and the use of project materials for research purposes. In accordance with the ethical principles of Photovoice (Hannes and Parylo, 2014), participants retained full autonomy over which photographs and narratives would be publicly displayed, -the extent of their personal identification, and their right to withdraw materials from public presentation at any stage. The examples presented in this article were selected with careful attention to participant confidentiality while balancing the need to protect participants with the need to illustrate the intervention model.

## 4 Illustrating the model: examples from the intervention process

The examples presented below were selected from the community intervention conducted in Ofakim following the events of October 7. They are based on a secondary analysis of the datasets collected throughout the project, including Photovoice units (photographs, titles, and narratives) and in-depth interviews with participants. Their purpose is not to present the findings of empirical studies based on these data, but rather to illustrate the core principles of TRIPVM and its implementation within a community intervention following a human-made collective disaster. Each example highlights a different component of the model. Taken together, they demonstrate how TRIPVM facilitates the identification, documentation, and mobilization of individual and community coping resources, while fostering movement from personal experience to a shared community narrative.

### 4.1 Example 1: reconstructing meaning in a traumatic space

Narrative (Voice):

“*This window, with its bars, silently witnessed the terror I experienced on October 7. My daughter and I found ourselves trapped behind these bars. I looked through this window in fear, hearing the sounds outside, barely daring to breathe. The bars that had always protected us suddenly felt like a prison. But today, when I look through the same window, I see something different. I see a community that came together, people reaching out to help one another. My volunteer work and participation in community events are like rays of light breaking through the bars. Every time I volunteer or take part in a community activity, I feel that I break another bar in my prison of fear. This window no longer reminds me only of fear; it also reminds me of the strength I discovered within myself and within my community. With each passing day, the bars become less of a barrier, and I can now see a future—a future in which we are stronger together and support one another.”*

Tsila describes the barred window of her home as a silent witness to the extreme fear and helplessness she experienced on October 7, when her home—a space traditionally associated with safety and protection—became a place of confinement and threat. Photographing the bars enables a renewed engagement with this symbol and opens the possibility of reinterpreting it (Figure 4). While the traumatic memory remains present, Tsila also identifies coping resources associated with the same image, including the solidarity of the community, volunteering, and participation in community activities.

**Figure 4:**
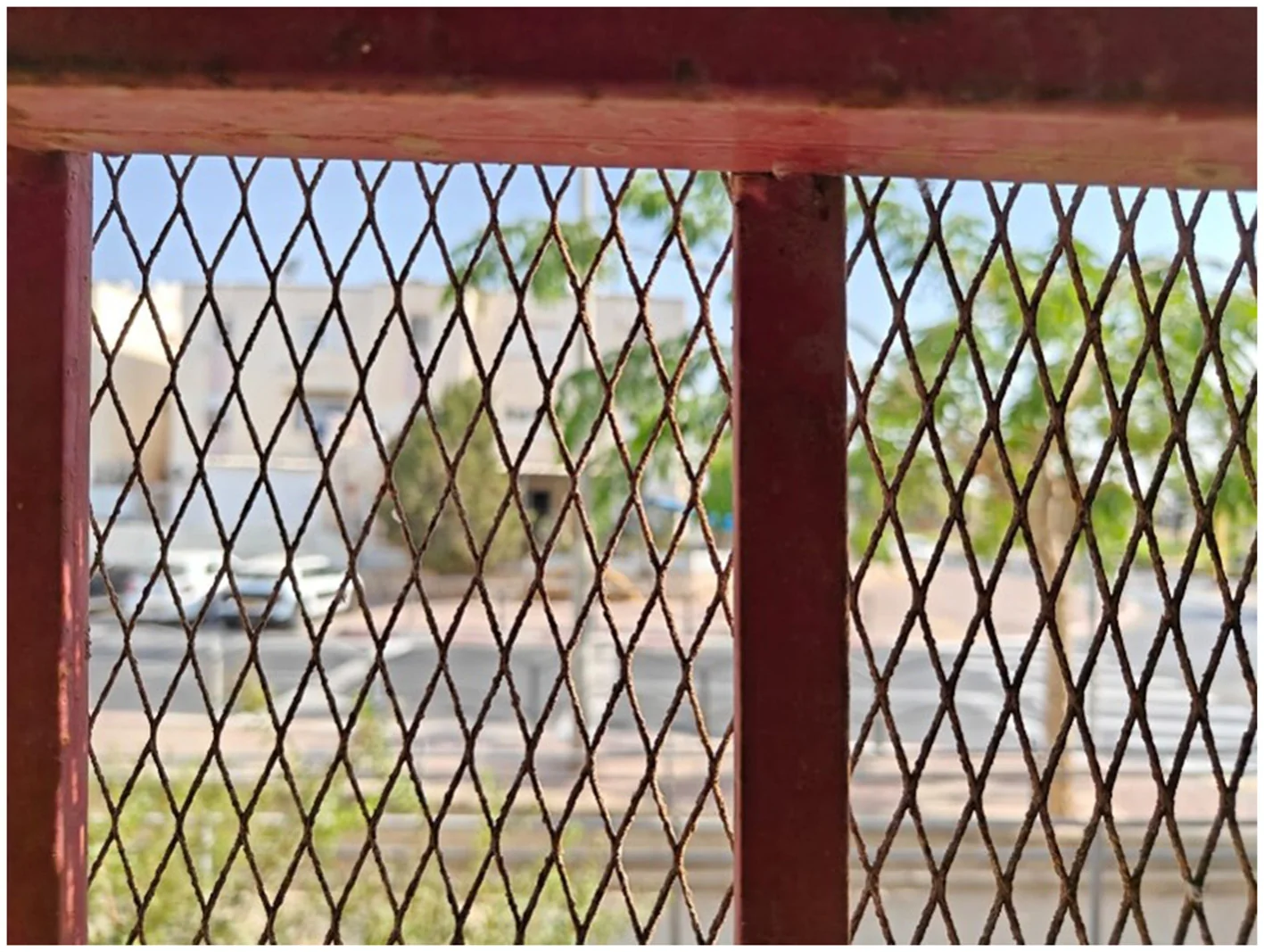
Breaking the Bars of My Prison.

Consequently, the bars come to embody two parallel meanings: one of danger and one of strength and possibility, represented by rays of light penetrating them. The ability to hold these meanings simultaneously reflects an expansion of meaning rather than the replacement of one meaning by another. This example illustrates one of TRIPVM's core principles, whereby resilience develops not through erasing traumatic memories but through expanding the meanings attributed to them and reconnecting them with individual and community coping resources.

### 4.2 Example 2: meaning embedded in place: Ofakim as a community

Narrative (Voice):

“*The photograph shows a green plant growing among weeds and debris on a boulevard near my home. I chose it because it tells the story of Ofakim—a city that has experienced immense pain - in its streets, pathways, homes, and in the hearts of its people. Yet within all of this, beauty slowly begins to grow. Life returns to the streets, people walk safely along the paths, damaged homes have been restored, and hope and faith are growing in people's hearts. May we continue learning from this remarkable city.”*

Dana chose to photograph a green plant emerging from a damaged environment, using it as a metaphor for Ofakim's story after the attack (Figure 5). Her narrative moves beyond the personal toward a collective account of the community, portraying a reality in which pain and destruction coexist with ongoing processes of recovery, renewed routine, and growth. By focusing on a green plant within a devastated landscape, she identifies coping resources that may remain present even when they are not the dominant features of the environment. Community solidarity is portrayed as something that develops from within the rupture itself.

**Figure 5:**
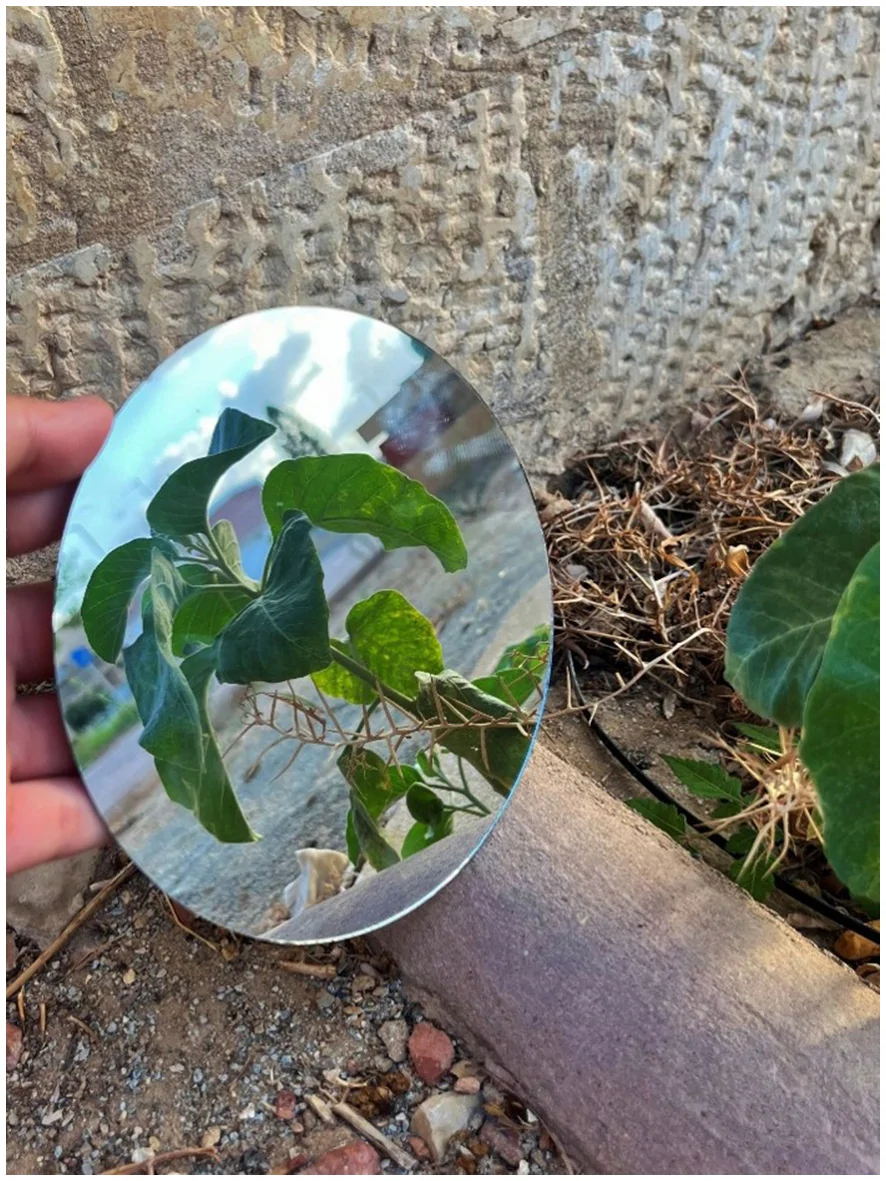
Beauty grows out of hardship.

Here again, trauma is not denied but becomes the background against which a resilience-oriented narrative is constructed. Moreover, this example demonstrates how positive representations of the community are transferred into the group setting, allowing other participants to engage with this alternative representation before it is further communicated to the wider public through the exhibition. This example illustrates how TRIPVM supports the development of community resilience narratives by transforming individual interpretations into collective representations of place and community.

### 4.3 Example 3: moving between self-states within a shared traumatic reality

Narrative (Voice):

“*The photograph shows several pairs of my shoes. As a social worker in the Ofakim Department of Social Services, I returned to work on October 8, 2023. Since then, like my colleagues, every morning I ask myself where my feet will take me today, and I wonder which shoes best fit the tasks ahead. Should I wear flats? Low heels? Sneakers? Or perhaps should I simply let my broken heart lead the way and stay in my slippers?”*

In this example, Hanna uses an ordinary everyday object—her shoes—to express the movement between her professional identity as a social worker and her identity as a resident of the affected community (Figure 6). Through Photovoice, new meanings are assigned to her work and that of her colleagues following October 7. She also conveys the challenge of carrying out her professional responsibilities while being exposed to the same traumatic reality as the people she serves. Coping resources emerge through the possibility of choosing among different pairs of shoes, each symbolizing a different professional role or task—for example, sneakers for fieldwork and home visits.

**Figure 6:**
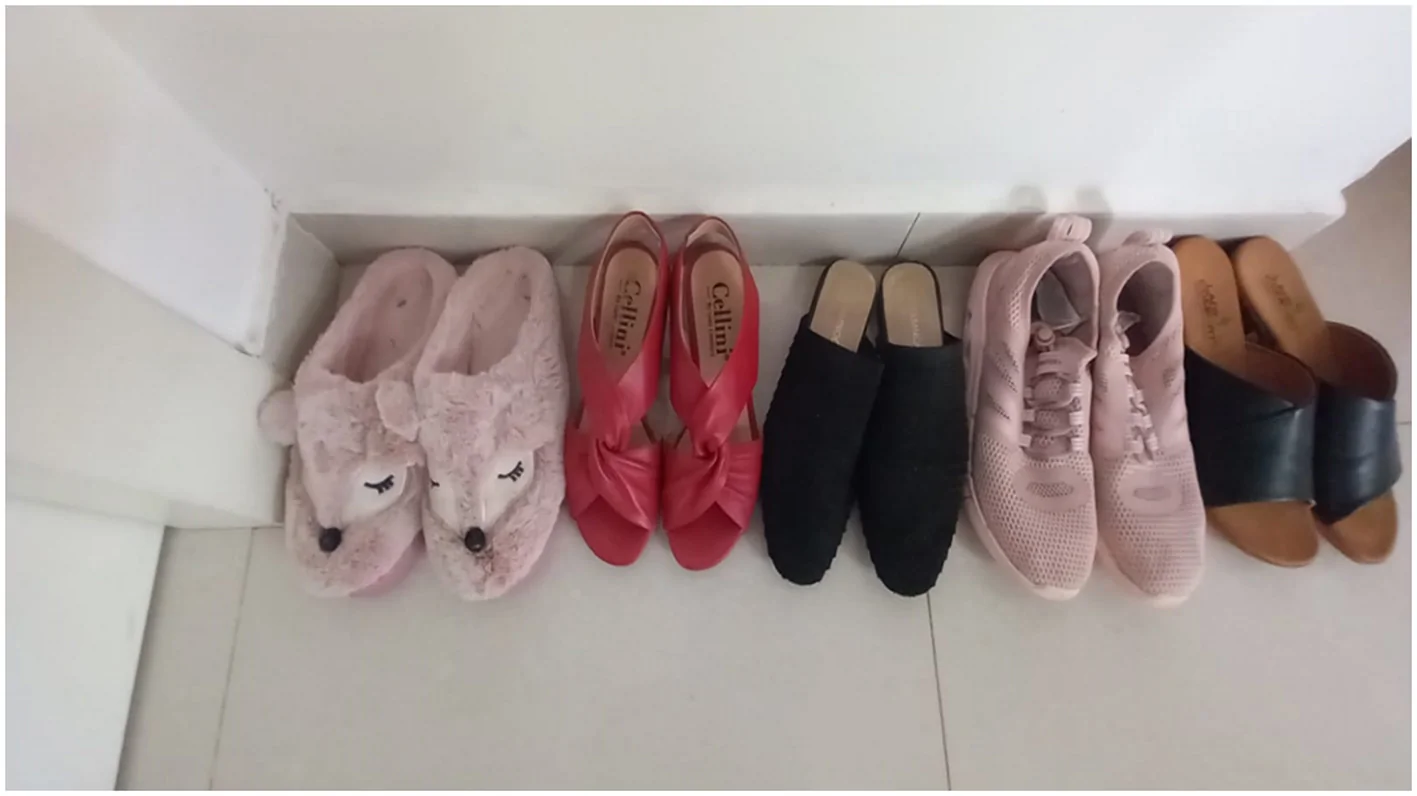
Should i let my broken heart lead the way?

The symbolic distinction between slippers, representing emotional paralysis and heartbreak, and the other shoes, representing action, purpose, and engagement, reflects the daily decision to leave home and continue serving the community. This example illustrates how TRIPVM enables participants to identify coping resources embedded within professional and social roles and to reconstruct the meaning of everyday professional practice within a shared traumatic reality.

### 4.4 Example 4: constructing social meaning through everyday action

Narrative (Voice):

*October 7 A table set, white tablecloths spread, Gunfire echoes, drops of wine are shed. Bodies scattered, sirens fill the air, A frozen body, tear-filled eyes, despair*.

*Day after day I sit and wonder, Is this reality, or only a dream? Should I sink into endless darkness, Or rise quickly and return to routine?*

*Without hesitation, one step after another, I stood firmly on my feet, Pushing the horror and massacre aside, Rolling up my sleeves for the soldiers, entering the kitchen. Whenever the memories resurface, I place another pot on the stove*.

Geula chose to express her experience through poetry, describing the tension between surrendering to the horrifying memories of October 7 and taking action. Ultimately, she chooses an everyday activity—cooking—which symbolizes not merely a return to routine but an act imbued with social meaning through volunteering and expressing gratitude toward soldiers (Figure 7). Cooking “for the soldiers” thus becomes both an act of community service and a source of personal healing. Rather than avoiding the traumatic memory, she repeatedly transforms it into purposeful action. This example illustrates TRIPVM's emphasis on action as a coping resource, demonstrating how ordinary daily practices can become meaningful vehicles for resilience, personal agency, and connection between individual experience and broader social context.

**Figure 7:**
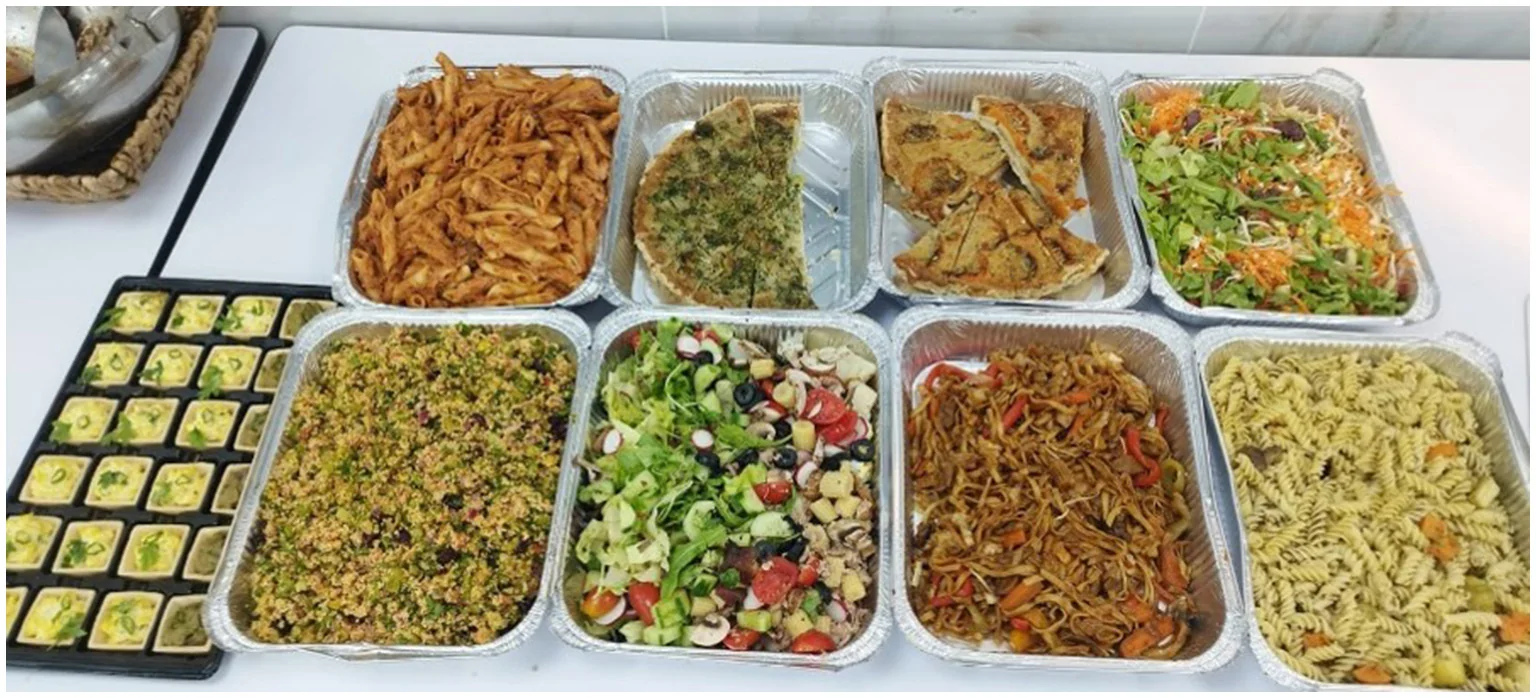
From Massacre to kitchen: a set table.

### 4.5 Example 5: memory, commemoration, and personal–community meaning

**Narrative (Voice):**

“*The photograph shows a tattoo that I received as a birthday gift. It reproduces my beloved brother's handwriting. Now he is engraved on my left arm, close to my heart, forever. All my life I heard about bereaved families, but only now do I truly understand what it means. I feel it like a knife through my heart—a hole that will never disappear. The tattoo itself did not hurt, because the emotional pain is far greater than the physical pain. This moment is also connected to my community because the tattoo artist shed a tear while working on my arm and said, ‘Your brother saved the neighborhood. This is the least I can do for your family.' I am waiting for every opportunity to continue telling my brother's story.”*

Haviva describes the tattoo as a reproduction of her brother's handwriting, transforming her own body into a living site of remembrance (Figure 8). By choosing to place the tattoo close to her heart, she gives expression to an unbearable grief that she expects to carry throughout her lifetime. The connection to the community emerges through the story surrounding the tattoo itself and her encounter with the tattoo artist, who reframes her brother's death through the language of heroism.

**Figure 8:**
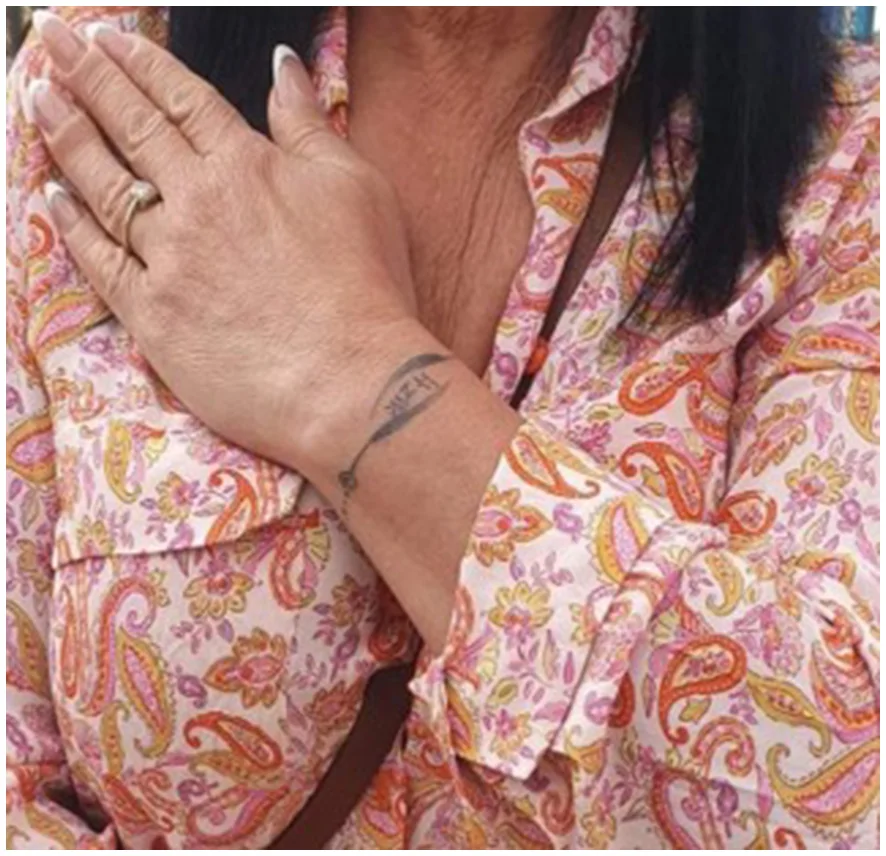
Engraved on my heart.

Recognizing him as someone who “saved the neighborhood” imbues her personal loss with collective significance, connecting private grief to a shared community narrative of remembrance. Rather than diminishing the pain, this recognition allows the loss to become part of an ongoing story that sustains memory and continuity. This example illustrates how TRIPVM integrates personal mourning with collective memory, demonstrating how resilience can emerge through the interplay of body, memory, recognition, and community.

### 4.6 Summary of the illustrations

The five examples presented above are not intended to represent the full range of experiences documented throughout the intervention. Rather, they were selected from the broader dataset because they illustrate different components of TRIPVM. Collectively, they demonstrate how the integration of photography, narrative writing, group dialogue, and public exhibition facilitates the identification, strengthening, and expansion of coping resources, supports the construction of resilience-oriented narratives, strengthens community ownership of its story, and enables movement from the “Story of Self” to the “Story of Us” (Ganz, 2009). These illustrations highlight TRIPVM's potential as a community-based intervention for fostering meaning-making, recognition, and resilience in the aftermath of a human-made collective disaster.

### 4.7 Discussion: TRIPVM as a community intervention following a collective disaster

The aim of this article was to present and examine TRIPVM as a community intervention following a human-made collective disaster, as developed and implemented within the broader intervention program conducted in the city of Ofakim after the events of October 7. To synthesize the article's main contributions, the discussion focuses on four central dimensions of the model: its positioning within a disaster management framework, its contribution as a narrative intervention promoting recognition, its capacity to identify and mobilize coping resources, and its potential for addressing trauma while reducing the risk of re-traumatization.

#### 4.7.1 Documenting, constructing, and disseminating resilience narratives

By situating TRIPVM within the broader disaster management framework (Figure 9), the model emphasizes the processing of disaster as a traumatic event through a continuum linking individuals, groups, and communities, while highlighting meaning-making, narrative construction, and movement from the “Story of Self” to the “Story of Us” (Ganz, 2009). This position is consistent with scholarship conceptualizing disasters not merely as material destruction but as disruptions of systems of meaning (Centemeri and Tomassi, 2022; Singh et al., 2026; Weik, 2008).

**Figure 9:**
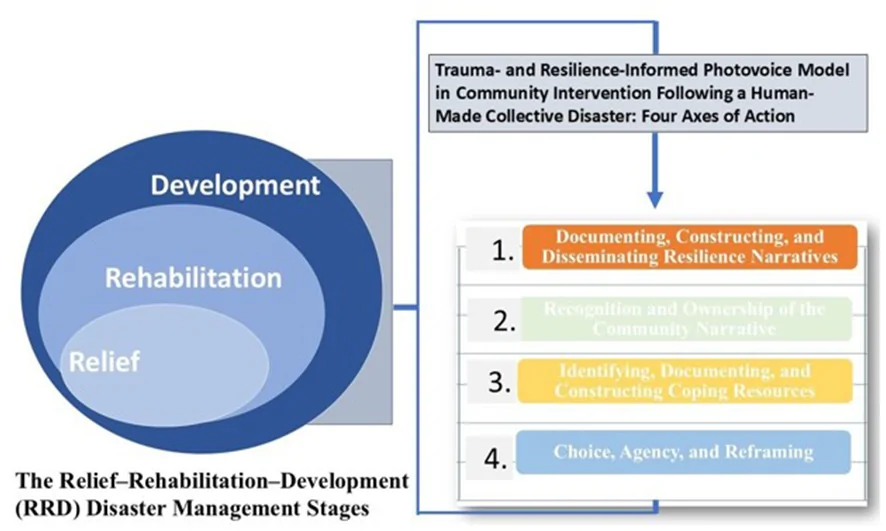
TRIPVM's four axes of action following a collective disaster.

Accordingly, even during the emergency relief phase—and particularly during the transition from relief to rehabilitation and development (Berke and Campanella, 2006; Christoplos et al., 2001)—the model creates opportunities for documenting, eliciting, constructing, and disseminating resilience narratives (Aung et al., 2021; Blit-Cohen, 2024; Ersing, 2020; Rawsthorne et al., 2023; Ratzon and Rappaport, 2023).

Earlier models have similarly linked community practice, narrative approaches, and participatory critical methodologies that promote dialogue, collaborative learning with communities, and the generation of meaningful knowledge translated into community narratives (Shahar and Shemer, 2012). The implementation of TRIPVM in Ofakim further underscores the importance of interventions explicitly aimed at producing, documenting, and disseminating resilience narratives. As suggested in the literature, privileging such narratives represents not only a professional choice but, in many respects, a political one: whether to reinforce trauma-centered discourse or, while fully acknowledging trauma and its consequences, facilitate the construction of alternative narratives emphasizing coping, agency, and resilience (Anderson et al., 2023; Catalani and Minkler, 2010; Imperiale and Vanclay, 2021; Maung et al., 2021).

Within the Israeli context, recent scholarship on community work in situations of disaster and war likewise emphasizes the importance not only of community-based interventions but, more specifically, of narrative-oriented interventions that facilitate community building, collective identity, and hope (Blit-Cohen, 2024). The integration of narrative with community practice also reinforces the understanding that responses to collective disasters cannot rely solely on individual psychological treatment. TRIPVM draws on the premise that when trauma is collective in nature, recovery likewise requires community-level interventions extending beyond the individual (Ersing, 2020; Itzhaki-Braun et al., 2023; Saul, 2022).

The illustrative examples presented throughout this article, representing only a small portion of the voices documented during the project, demonstrate the importance of community intervention centered on resilience narratives. In Example 1, participation in community volunteering and collective activities serves as a counterweight to the overwhelming effects of traumatic exposure. Similarly, Example 4 highlights volunteering through cooking for soldiers, while Example 5 illustrates how attributing social meaning to the death of a community member—as someone who saved others—transforms personal loss into a source of collective significance. Together, these examples demonstrate how community interventions create opportunities to negotiate meanings within the very social spaces where the disaster occurred (Flores et al., 2025).

#### 4.7.2 Recognition and ownership of the community narrative

Beyond its narrative orientation, TRIPVM is grounded in the critical foundations of Photovoice, particularly its sensitivity to power relations and democratization of knowledge production (Catalani and Minkler, 2010; Wang and Burris, 1997). Accordingly, the intervention incorporates an additional dimension: fostering recognition and strengthening the community's ownership of its representations within the broader social, cultural, and public sphere (Maciel and Sellnow, 2025; Mosley and Biernat, 2025).

In the case of Ofakim—a peripheral community historically associated with limited symbolic capital and negative representations within Israeli narratives of development towns (Cohen, 2016; Gigi and Tzfadia, 2024)—this meant creating a space in which residents could negotiate, construct, and disseminate alternative narratives about their city and community (Malka, 2024). The public exhibition, conceived as an integral component of the intervention rather than its final product, played a central role in this process by facilitating movement from the protected group setting into the public sphere, where community narratives could enhance visibility, validate lived experiences, and contribute to the construction of collective memory and social meaning (Bray et al., 2023; Frankenburg and Org, 2025; Padilla et al., 2024; Stone and Grebenar, 2022).

This understanding resonates with (Taylor 1994) concept of recognition, according to which psychological recovery following traumatic events is inseparable from broader processes of public recognition and social positioning. Recognition enables communities to challenge dominant representations and to establish alternative narratives within wider public and institutional discourse. Conversely, misrecognition—or non-recognition (Taylor, 1994)—may itself constitute a source of psychological suffering by shaping collective identities through exclusion and symbolic marginalization, thereby reinforcing the toxic consequences of disaster and trauma.

Importantly, the need for recognition should not be viewed as unique to communities already experiencing social marginalization. The experience of collective disaster itself places communities in a condition of vulnerability (Bonanno et al., 2010; Milošević, 2024; Van Dorn et al., 2020), making questions of recognition, misrecognition, and non-recognition (Taylor, 1994) highly relevant regardless of their prior social status. In Ofakim, this need was reflected not only in the participants‘ collective decision to title the exhibition Our Story, Ofakim, but also in their repeated efforts to portray the city's strengths and resources. Example 2 clearly illustrates this process by emphasizing residents' efforts to represent Ofakim as a city characterized by courage, solidarity, and resilience rather than solely by victimhood (Malka, 2024).

#### 4.7.3 Identifying, documenting, and constructing coping resources

TRIPVM demonstrates how both the adapted group facilitation process and the modified narrative protocol systematically guide dialogue at the individual, group, and community levels toward the identification, documentation, and mobilization of coping resources. This approach is grounded in the Conservation of Resources (COR) Theory (Hobfoll, 2001), which posits that coping with stress and trauma depends on individuals' ability to preserve, mobilize, and develop resources, particularly under conditions characterized by the threat or actual loss of resources, as is often the case following disasters.

The examples presented throughout this article illustrate how TRIPVM facilitates both the identification of existing resources and the ways in which these resources acquire meaning in everyday life. In the example “Breaking the Bars of My Prison of Fear,” community engagement and volunteering function as social resources, fostering belonging, mutual support, and the investment of time and effort in meaningful collective action, thereby enabling movement from feelings of confinement toward purposeful engagement. In the example of the green plant growing among the ruins, personal resources are reflected in the participant's capacity to construct meaning and identify hope within an adverse reality. Similarly, the example of the different pairs of shoes illustrates psychological flexibility and adaptability as important personal resources. In the example of cooking for soldiers, everyday action itself becomes an energetic and motivational resource, while the tattoo represents an object resource carrying both personal memory and collective recognition of loss. Together, these examples demonstrate participants' agency in identifying, mobilizing, and strengthening coping resources, while assigning new meanings to them through the Photovoice process (Dixon et al., 2022; Olausson and Lindahl, 2022; Pichon et al., 2022; Rolbiecki et al., 2016).

Importantly, within TRIPVM, coping resources are not understood as static personal attributes. Rather, they emerge through a dynamic interaction between individuals, their communities, and the meanings they collectively construct. In this sense, the intervention extends beyond identifying existing strengths; it actively contributes to strengthening and expanding psychological and social resources through dialogue, reflection, and public recognition.

#### 4.7.4 Choice, agency, and reframing

Finally, the proposed TRIPVM offers a distinctive application of several principles closely associated with trauma- and resilience-informed practice. Its uniqueness lies in the deliberate integration of photography, narrative writing, structured group facilitation, and carefully managed public dissemination. Collective disasters are frequently associated with experiences of overwhelming helplessness, loss of control, and the consolidation of negative meanings surrounding traumatic events (Heanoy and Brown, 2024; Weik, 2008). In response, TRIPVM creates multiple opportunities for participants to exercise choice: what to photograph, how to frame reality, how to interpret their experiences, and ultimately what to share with others. The very act of choosing constitutes an important therapeutic and community process, restoring a sense of agency within circumstances largely experienced as uncontrollable (Anderson et al., 2023).

As illustrated by the examples presented in this article, TRIPVM facilitates processes of reframing traumatic experiences and their consequences. Participants deliberately chose to photograph ordinary everyday objects—a barred window, a small plant, different pairs of shoes, cooking utensils, or a tattoo—and, through narrative reflection, transformed these objects into symbols carrying meanings associated with resilience, hope, belonging, and continuity. Rather than denying the traumatic event or minimizing its consequences, the intervention enables participants to construct alternative meanings that coexist alongside traumatic memories. In doing so, TRIPVM promotes an interpretive movement that neither erases nor replaces trauma but expands the range of meanings available to individuals and communities. Such meaning-oriented reconstruction has been identified as a central mechanism in resilience building following collective trauma (Olausson and Lindahl, 2022; Singh et al., 2026; Weik, 2008).

The emphasis on choice extends beyond the individual level. Participants also exercise agency collectively by deciding which stories will represent their community, which photographs will be publicly exhibited, and how their community wishes to be remembered. Consequently, the intervention supports not only individual empowerment but also collective agency, enabling communities to shape their own public narrative following disaster.

## 5 Conclusion

The model presented in this article positions Photovoice as a structured community intervention embedded within the rehabilitation phase following a collective disaster. It integrates four complementary components: disaster management, resilience narratives, recognition and ownership of the community story, and the identification and mobilization of coping resources. The model's distinctive contribution lies in moving beyond documenting traumatic experiences toward creating conditions for choice, meaning-making, public recognition, and collective ownership. In doing so, it enables communities to participate actively in constructing the meaning of disaster and in shaping continuity and recovery in its aftermath.

From a theoretical perspective, TRIPVM extends the traditional application of Photovoice by integrating principles drawn from disaster management, trauma- and resilience-informed practice, and community narrative approaches. Rather than conceptualizing Photovoice primarily as a participatory research methodology, the model positions it as a structured community intervention designed to facilitate recovery following human-made collective disasters. In this respect, TRIPVM contributes to the existing literature by demonstrating how the methodology can be systematically adapted to contexts characterized by collective trauma while preserving its critical and participatory foundations.

From a practical perspective, the model offers professionals a structured framework for implementing community interventions during the transition from emergency response to rehabilitation. These adaptations—including the modified SHOWeD protocol, structured facilitation principles, the emphasis on coping resources, and the integration of a public exhibition—provide concrete guidelines for translating trauma- and resilience-informed principles into community practice. Although developed in the context of Ofakim following the October 7 attacks, these principles may be applicable to additional contexts involving communities affected by war, terrorism, natural disasters, or other collective crises.

At the same time, several limitations should be acknowledged. First, the present article focuses primarily on presenting the conceptual model, its underlying principles, and its implementation, rather than systematically examining participants' experiences or evaluating the intervention's effectiveness. Although the illustrative examples are drawn from datasets that were analyzed through separate empirical studies, the article does not report those findings. Future research should therefore examine the model's contribution from the perspectives of participants, facilitators, and community stakeholders, as well as its longer-term influence on community resilience and recovery.

Second, the article does not address processes of formal program evaluation, implementation fidelity, or lessons learned by the professional teams responsible for delivering the intervention. These issues warrant further investigation, particularly regarding the transferability of TRIPVM across cultural contexts, disaster settings, and community populations.

Despite these limitations, TRIPVM offers a theoretically grounded and practice-oriented framework for community intervention following collective disasters. By integrating narrative construction, community participation, recognition, public engagement, and the identification of coping resources, it expands the role of Photovoice beyond participatory research and positions it as a meaningful approach to supporting collective recovery, restoring agency, and strengthening community resilience in the aftermath of trauma.

## Data Availability

The raw data supporting the conclusions of this article will be made available by the authors, without undue reservation.
